# Enhanced antioxidant activity and quality of olecranon peach fruits (*Prunus persica* L.) through synergistic application of exogenous nano-selenium and melatonin

**DOI:** 10.1007/s44297-023-00017-6

**Published:** 2023-12-05

**Authors:** Peijuan Miao, Qinyong Dong, Chunran Zhou, Dong Li, Huan Yu, Yongxi Lin, Yangliu Wu, Canping Pan

**Affiliations:** 1 Key Laboratory of Tropical Fruits and Vegetables Quality and Safety for State Market Regulation, College of Science, China Agricultural University, Haikou, 570311 China; 2 Key Laboratory of Green Prevention and Control of Tropical Plant Diseases and Pests, Ministry of Education, College of Plant Protection, Hainan University, Haikou, Hainan 570228 People’s Republic of China

**Keywords:** Olecranon peach, Nano-selenium, Melatonin, Quality, Antioxidant ability

## Abstract

Olecranon peach, a significant economic crop in southern China, faces quality degradation due to pests, diseases, and pesticide residues. Melatonin (MT) and nano-selenium (Nano-Se) applied individually have been found to improve crop growth and abiotic stress resistance. However, the impact of different Nano-Se and MT ratios on olecranon peach quality remains uncertain. Plants were foliar sprayed with varying Nano-Se and MT ratios during the fruit set phase. A combined 5 mg/L Nano-Se and 10 mg/L MT (Nano-Se5 + MT10) effect outperformed individual treatments and the control in enhancing fruit quality. Nano-Se5 + MT10 notably increased peroxidase (190.3%) and superoxide dismutase (112.2%) activities, and reduced polyphenol oxidase activity (-13.2%), raised total sugars (33.3%), soluble protein (18.5%), and phenolic contents like apigenin (51.9%), vanillic acid (21.4%), ferulic acid (29.4%), and *p*-hydroxybenzoic acid (317.2%) compared to the control. Correlation analysis linked antioxidant enzymes and phenolics in olecranon peach fruits to nutrient accumulation. Nano-Se5 + MT10 improves sweetness and quality by enhancing antioxidant ability (increased peroxidase and superoxide dismutase, decreased polyphenol oxidase and phenolics), and promoting soluble protein, total sugar, and phenolic accumulation. In conclusion, Nano-Se5 + MT10 application shows promise in enhancing olecranon peach quality and antioxidant attributes.

**Graphical Abstract:**

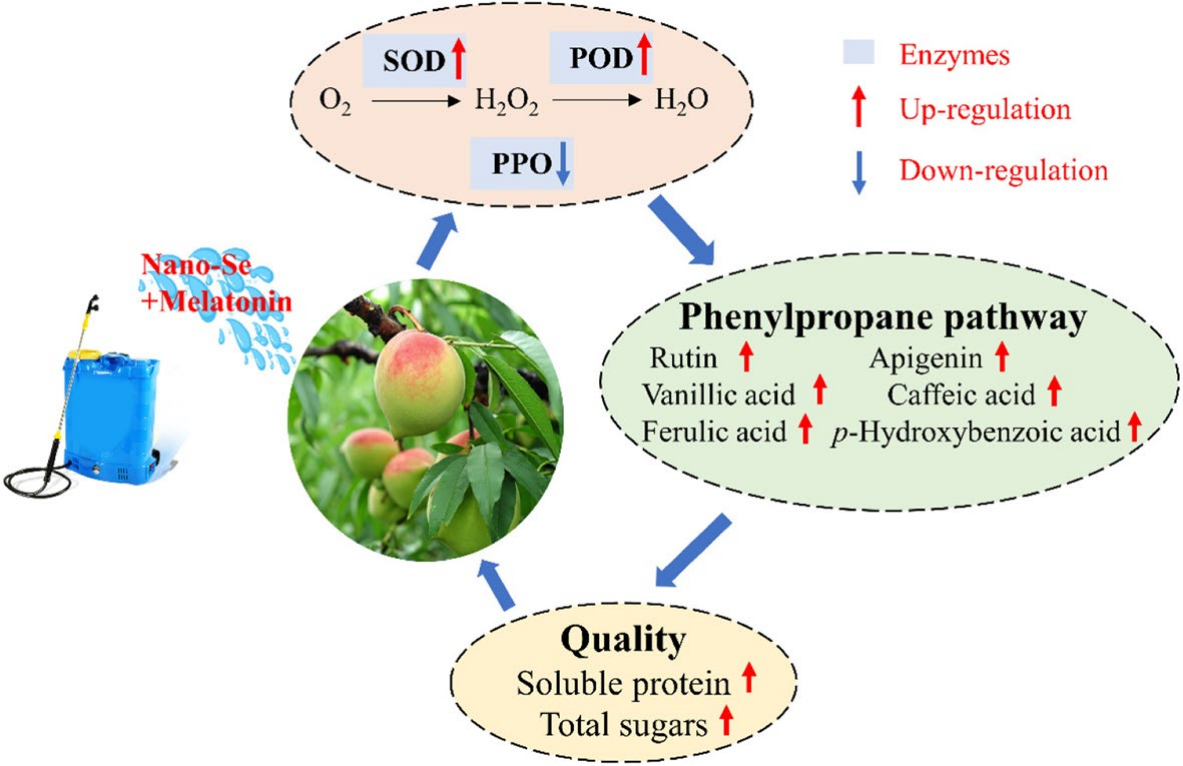

**Supplementary Information:**

The online version contains supplementary material available at 10.1007/s44297-023-00017-6.

## Introduction

Olecranon peaches (*Prunus persica* L.), which are produced in Guangdong Province, China, are distinctive on the market for their vibrant color, crisp texture, and honey-like sweetness. The annual supply can barely meet the ever-growing demand [1]. However, while it is growing and developing, olecranon peach is subject to several environmental challenges, including low temperatures, drought, hot temperatures, high salinity, and pesticide exposure [2]. These stresses can cause fruit to dehydrate, wilt, soften, or even decay, all of which have a negative impact on the fruit’s quality and post-harvest storage, severely reducing both its edibility and marketability. To meet the rising market demand for olecranon peaches, it is crucial to investigate practical agricultural approaches to improve the quality of olecranon peaches and reduce the negative effects of environmental pressures during production.

In recent years, due to their safety and non-toxic qualities, exogenous biological stimulants have drawn scientific interest as a way to guarantee the quality of fruits and vegetables. Plant hormones and their analogs, biopolymers, and nanoparticles are the main types of biostimulants [3–5]. Melatonin (MT), also known chemically as N-acetyl-5-methoxytryptamine, was initially identified as an indole-class neurohormone released by the pineal gland of mammals. It is renowned for its anti-inflammatory effects, anti-aging benefits, sleep-improving qualities, and therapy of neurological illnesses [6]. Numerous studies have been conducted since Dubbels et al. [7] discovered the presence of MT in plant tissues in 1995 have shown that MT plays a role in controlling a variety of physiological processes in plants, including growth, development, photosynthesis, rooting, seed germination, and responses to biotic and abiotic stresses [8–13]. Additionally, MT can act directly as an antioxidant and an endogenous free radical scavenger. Studies have shown that MT could reduce the production of post-harvest fruit malondialdehyde (MDA), hydrogen peroxide (H_2_O_2_), superoxide anion (O^2−^), and polyphenol oxidase (PPO). Additionally, it increases the activity of the following antioxidant enzymes: catalase (CAT), superoxide dismutase (SOD), peroxidase (POD), ascorbate peroxidase (APX), and phenylalanine ammonia-lyase (PAL) [14]. These outcomes aid in lowering post-harvest oxidative damage and maintaining post-harvest storage quality. The quality and antioxidant content of fruits, including sugars, ascorbic acid, polyphenols, and flavonoids, have also been demonstrated to be impacted by exogenous MT administration, according to a previous study [4, 15, 16]. With the application of nanotechnology in agriculture maturing, the use of nano fertilizers to enhance crop quality has received extensive research [17]. Selenium is an essential trace non-metallic element for the human body [18]. Nano-selenium (Nano-Se) is a safer option for the production of selenium-enriched plants since its acute toxicity is seven times lower than that of sodium selenite [19]. It has been demonstrated that 20 mg/L Nano-Se intervention enhances the accumulation of capsaicin, nutrients, and secondary metabolites in chili peppers, thereby improving the antioxidant capacity and nutritional quality of chili pepper fruits [20]. Research on tomatoes also revealed that 1 mg/L Nano-Se can improve tomato growth and flavor quality by modulating plant hormones, sugars, acids, and volatile compounds [21]. Additionally, Kang et al. discovered that Nano-Se treatment increases the resistance of melon plants to powdery mildew by boosting antioxidant capacity and photosynthesis, and maintaining a steady-state equilibrium between the production and scavenging of reactive oxygen species [22].

Based on multiple studies, the exogenous application of Nano-Se and MT provides a promising strategy for resolving the quality and health issues of agricultural products. However, to date, there is no existing research reporting the effects of Nano-Se and MT individually or in combination on the quality of olecranon peaches. This study intends to investigate the likely processes that enhance the quality of olecranon peaches following foliar treatment with Nano-Se and MT. The antioxidant enzymes, quality components, and phenolic compounds were evaluated under Nano-Se and MT intervention. The ideal levels of Nano-Se and MT were identified after thorough analysis, and it was shown that their combined effects on olecranon peach quality were synergistic. This information provides technical support and evidence for Nano-Se and MT rational application and quality enhancement in olecranon peach production.

## Materials and methods

### Chemicals

Chromatography-grade acetonitrile and formic acid were obtained from Fisher Scientific (Beijing, China). Anhydrous ethanol was sourced from Beijing Chemical Plant Co., Ltd. (Beijing, China). Nano-Se was provided by Guilin Jiqi Biochemical Co., Ltd. (Guilin, China). Melatonin (MT; > 98%) was obtained from Shanghai Maclin Technology Co., Ltd. (Shanghai, China). Octadecylsilane (C18, 40 mm) was obtained from HAMAG Instrument Technology Co., Ltd. (Zhejiang, China). All standards were purchased from Yuanye Bio-Technology Co. Ltd. (Shanghai, China).

### Field experiment

The olecranon peaches were sourced from the orchard in Qinggang Village, Bao’an Town, Qingyuan City, Lianzhou, Guangdong Province, China (112°21'E, 24°53'N). Five treatment groups and one control group made up a total of six groups in the experiment, with five peach plants in each group serving as five repetitions. The spraying concentrations of Nano-Se and MT were set based on our previous field experiments and laboratory studies [23–25]. The treatment groups were as follows. (1) 5 mg/L nano-selenium (Nano-Se5); (2) 10 mg/L nano-selenium (Nano-Se10); (3) 10 mg/L melatonin (MT10); (4) 5 mg/L nano-selenium + 10 mg/L melatonin (Nano-Se5 + MT10); (5) 10 mg/L nano-selenium + 10 mg/L melatonin (Nano-Se10 + MT10). Equal water amounts were sprayed on the control group. A month before olecranon peaches ripened, the leaves were sprayed with Nano-Se and MT once every 7 days for a period of 3 weeks. Fruit samples were taken seven days after the completion of the three-week treatment period from various locations on the tree and guaranteed minimal damage, uniform size, and constant freshness. All samples were thoroughly cleaned with distilled water to ensure that no exogenous chemicals were present before measuring antioxidant enzymes and metabolites, and they were then quickly frozen at -80 °C.

### Determination of antioxidant capacity

POD, SOD, MDA, DPPH, and PPO were identified using detection kits. The main steps were as follows. Fresh olecranon peach fruits were powdered in liquid nitrogen, and 0.1 g of the powder was then added to 1 mL 0.1 mol/L phosphate-buffered solution (PBS, pH 7.0–7.4) and vortexed for 3 min. The homogenate was centrifuged at 4 °C for 10 min at a speed of 5000 rpm, and the supernatant was utilized to detect antioxidant-related indicators. Utilizing the guaiacol colorimetric technique, POD activity was assessed. The nitroblue tetrazolium (NBT) method was used to gauge the activity of SOD. MDA levels were assessed using the thiobarbituric acid reactive substances (TBARS) assay. The 1,1-diphenyl-2-picrylhydrazyl (DPPH) test was used to calculate the total antioxidant capacity. Using a catechol colorimetric technique, PPO activity was determined. All assays were performed using reagent kits from Suzhou Koming Biotechnology Co., Ltd. (China).

### Determination of nutrients

Soluble sugar, soluble protein, total sugar, and ascorbic acid assay kits were purchased from Suzhou Koming Biotechnology Co., Ltd. (China). The determination of soluble sugar content was performed using the anthrone colorimetric method. Using the Coomassie Brilliant Blue G-250 staining technique, the amount of soluble protein was determined. The total sugar content in peach fruits was determined using the 3,5-dinitrosalicylic acid (DNS) method.

### Analysis of phenolic compounds

The determination of phenolic compounds in peaches was conducted following the method described by Li et al. [26], with slight modifications. Briefly, 100 mg of peach fruits were powdered under liquid nitrogen. They were poured into a 2 mL centrifuge tube along with 1 mL of water that contained 60% ethanol. The solution was ultrasonically processed for 30 min at 30 °C, followed by a 2-min centrifugation at 12,000 rpm. By repeating the operating process two times, the extract was obtained. The mixture was collected, nitrogen-blow-dried, fixed to 1 mL of 60% ethanol, and purified with 100 mg of C18 in a 2 mL centrifuge tube. The purified mixture was then shaken for 2 min and centrifuged for 1 min at 10,000 rpm. Next, a 0.22 μm nylon syringe was used to filter the supernatant into the injection vial. The Agilent 6410B Triple Quadrupole HPLC–MS/MS (Agilent Technologies, U.S.) was fitted with an HPLC reversed-phase C18 column (Athena C18-WP 2.1 × 50 mm, 3 μm) for the analysis of the concentration. The parameters are shown in Tables S1 and S2.

### Statistical analysis

One-way analysis of variance (ANOVA) was used to compare various treatments using SPSS 26.0 (IBM, Inc., Armonk, NY, USA), and statistical significance was established using Duncan’s test at *p* < 0.05. Origin Pro 2022 (Northampton, Massachusetts, USA) was used to make the graphs.

## Results

### Effects of combined application of Nano-Se and MT on antioxidant capacity in olecranon peach fruits

**Fig. 1:**
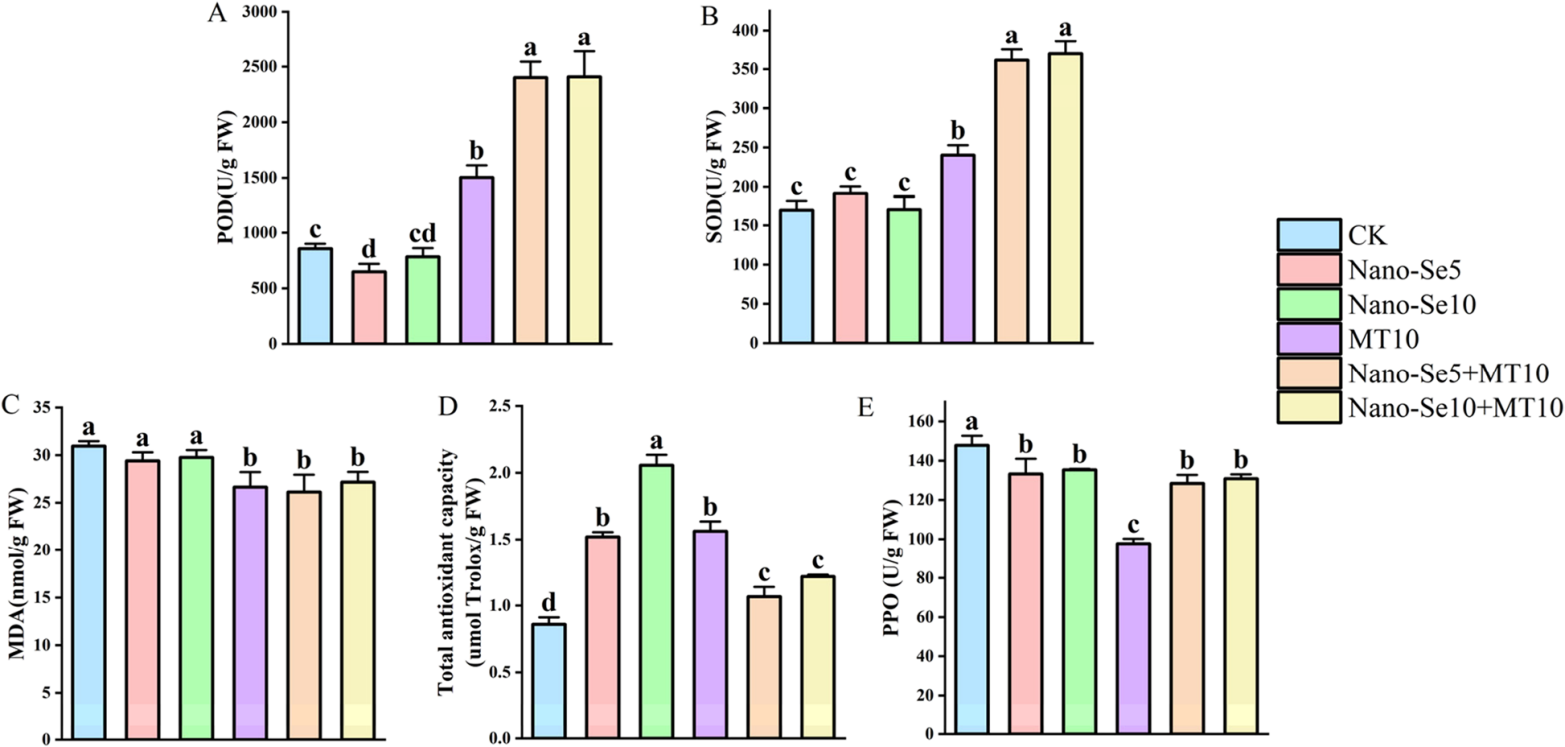
Comparison of POD (**A** U/g FW), SOD (**B** U/g FW), MDA (**C** nmol/g FW), Total antioxidant capacity (**D** μmol Trolox/g FW), and PPO (**E** U/g FW) levels in olecranon peaches treated with various concentrations of Nano-Se and MT. Different letters show statistically significant differences when *p* < 0.05

POD and SOD are crucial antioxidant enzymes in the enzyme-mediated scavenging system, playing a key role in preventing oxidative damage from H_2_O_2_ and O_2_^−^ and in maintaining the equilibrium of reactive oxygen species (ROS) metabolism [27]. Compared to the control, the combined treatment exhibited better enhancement in antioxidant enzyme activities than the individual treatments. As shown in Fig. 1A and B, POD activity in the Nano-Se5 + MT10 treatment group was 300.0% and 52.5% higher than that in the Nano-Se5 and MT10 treatment groups, respectively. SOD activity increased by 88.6% and 50.3%, respectively. Similarly, compared to the Nano-Se10 and MT10 treatment groups, POD activity in the Nano-Se10 + MT10 treatment group increased by 219.3% and 54.2%, and SOD activity increased by 116.1% and 53.6%, respectively. MDA is considered one of the major lipid peroxides and primarily reflects the degree of membrane lipid peroxidation induced by reactive oxygen species [27]. Compared with the control group, the MDA content in the Se5 + MT10 and Se10 + MT10 treatments was significantly reduced by 15.6% and 12.2%, respectively (Fig. 1C). PPO is a key oxidative enzyme that causes phenolic substances in fruits to oxidize and leads to the browning of the fruit pulp [28]. Compared to the control, all treatment groups exhibited significantly reduced PPO activity. Specifically, the Nano-Se5 + MT10 and Nano-Se10 + MT10 treatments resulted in 13.2% and 11.5% decreases in PPO activity, respectively (Fig. 1E). Additionally, the Nano-Se5 + MT10 and Nano-Se10 + MT10 treatment groups had total antioxidant capacities that were 24.1% and 41.7% higher, respectively, than those of the water control group (Fig. 1D). In summary, olecranon peach fruit total antioxidant capacity was significantly increased by nano-selenium and melatonin interventions, and PPO activity was suppressed. However, compared to the Nano-Se5, Nano-Se10, and MT10 treatment groups, the Nano-Se5 + MT10 and Nano-Se10 + MT10 treatment groups showed significantly higher POD and SOD activities, demonstrating their superior capability in eliminating excess free radicals and enhancing the stress resistance of olecranon peach.

### Effect of Nano-Se, MT and their combination on nutrients in olecranon peach fruits

**Fig. 2:**
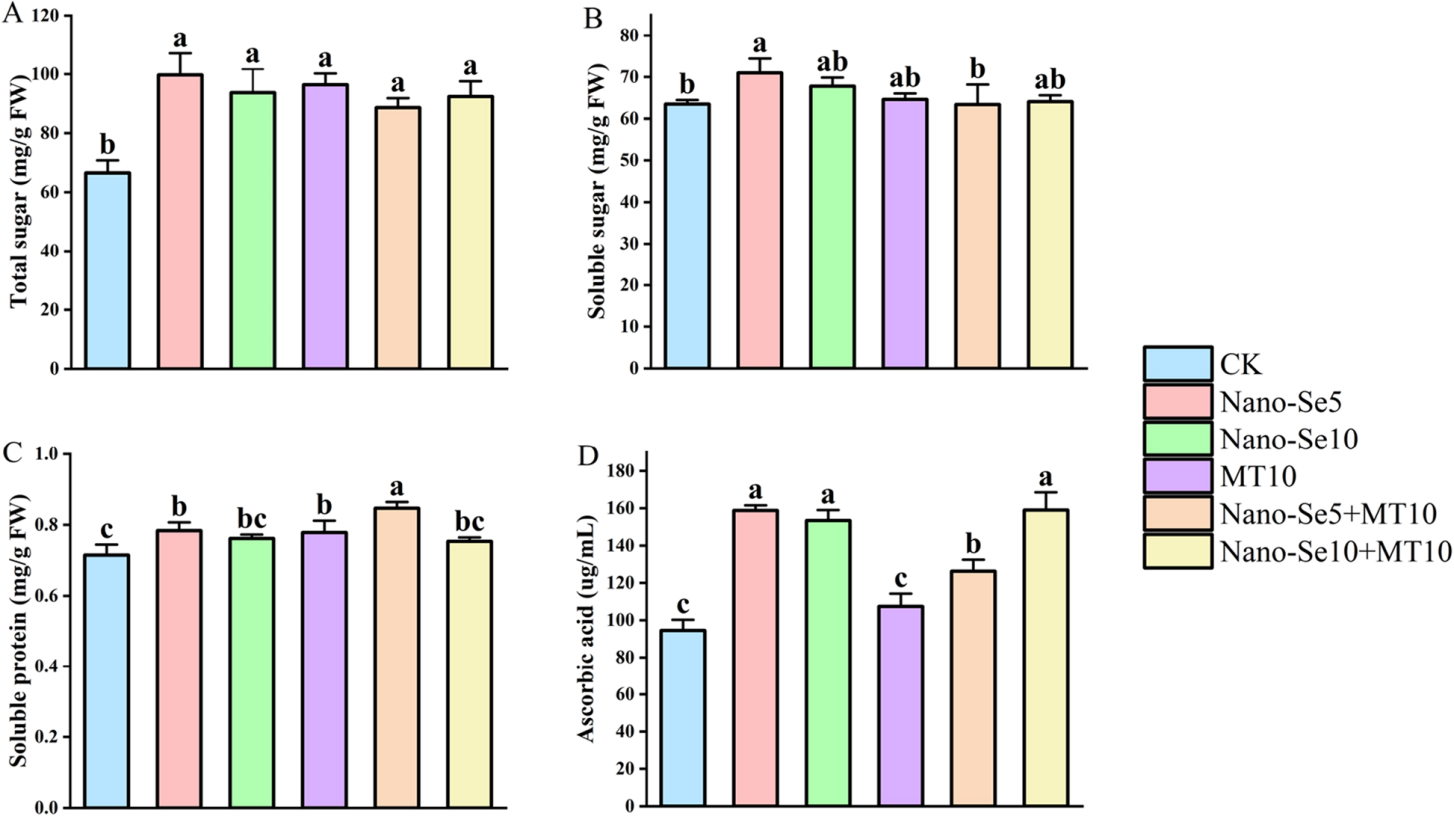
Total sugar (**A** mg/kg FW), Soluble sugar (**B** mg/kg FW), Soluble protein (**C** mg/kg FW), and Ascorbic acid (**D** μg/mL) levels in olecranon peaches were affected by varied Nano-Se and MT treatment ratios. Differences that are statistically significant at *p* < 0.05 are shown by different letters

Fruit quality encompasses attributes such as appearance, internal characteristics, and safety aspects. Internal quality reflects the combined expression of biochemical properties, flavor, and nutritional value, serving as a crucial indicator of fruits’ commercial viability; it includes the evaluation of parameters such as sugars, proteins, and vitamins [29]. In this study, we found that the intervention of exogenous Nano-Se and MT could enhance the nutritional quality of olecranon peach, as shown in Fig. 2. All treatment groups exhibited varying degrees of improvement in total sugar, soluble sugar, soluble protein, and ascorbic acid contents of olecranon peach. Specifically, Fig. 2A and B demonstrate that all treatment groups significantly increased the total sugar content of olecranon peach compared with CK. The total sugar content of the Nano-Se5 + MT10 and Nano-Se10 + MT10 treatment groups increased by 33.3% and 39.0%, respectively, and the combined treatments also had some promotive effects on soluble sugar accumulation, although the impact was not statistically significant. Exogenous Nano-Se and MT interventions increased the levels of soluble protein compared to CK, as shown in Fig. 2C. Among the treatment groups, the Nano-Se5 + MT10 group exhibited the highest accumulation of soluble protein, surpassing the Nano-Se5, MT10, and Nano-Se10 + MT10 treatments by 8.1%, 8.8%, and 12%, respectively. This indicated that exogenous interventions with Nano-Se and MT could enhance the soluble protein content of olecranon peach, thereby increasing its nutritional value. The combination of Nano-Se5 + MT10 exhibited a synergistic effect on the accumulation of soluble protein in fruits. Figure 2D illustrates that all treatment groups, with the exception of the MT10 treatment group, were able to significantly increase the ascorbic acid content of the fruit. Of the three different treatment groups (Nano-Se5, Se10, and MT10), the effect of the Nano-Se5 treatment group on ascorbic acid accumulation in olecranon peach was greater than that of the Nano-Se10 and MT10 treatment groups by 3.6% and 47.8%, respectively. It was indicated that Nano-Se5 treatment group could be the best single exogenous treatment to enhance the ascorbic acid content in olecranon peach compared to the Nano-Se10 and MT10 treatment groups. In addition, the ascorbic acid content of the Nano-Se5 + MT10 and Nano-Se10 + MT10 treatment groups were elevated by 33.7% and 68.2% compared to CK, respectively, which were 17.6% and 48.0% higher than that of the MT10 treatment group.

### Effect of Nano-Se, MT and their combination on phenolics in olecranon peach fruits

**Fig. 3:**
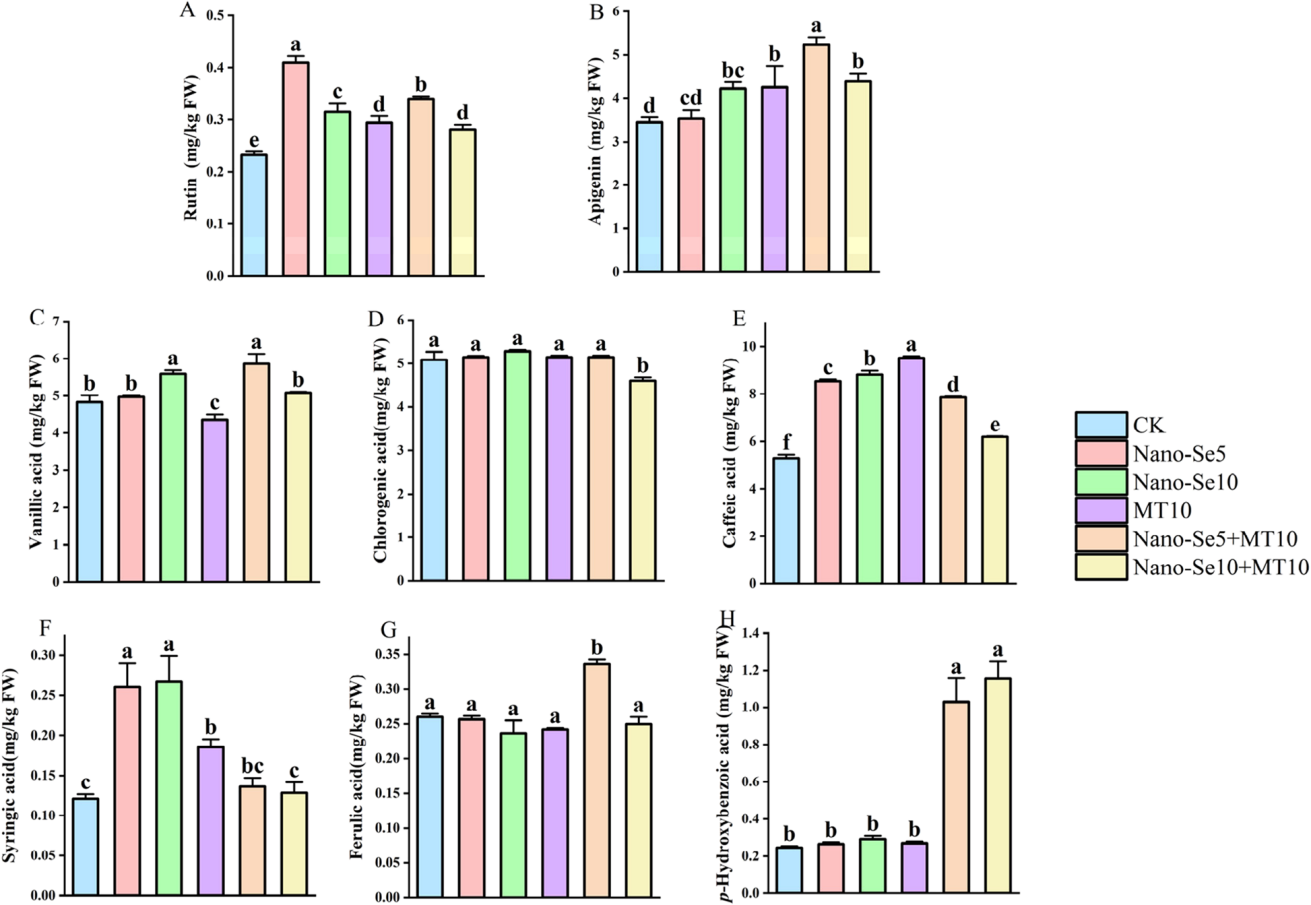
Olecranon peach levels of Rutin (**A**), Apigenin (**B**), Vanillic acid (**C**), Chlorogenic acid (**D**), Caffeic acid (**E**), Syringic acid (**F**), Ferulic acid (**G**), and* p*-Hydroxybenzoic acid (**H**) as a result of spraying Nano-Se and MT in varied changes that are statistically significant at *p* < 0.05 are shown by distinct letters

Fruits and vegetables typically require the involvement of enzyme systems and antioxidant molecules to preserve optimum quality [30]. Under a variety of abiotic stressors, phenolics, essential non-enzymatic antioxidants in plants, can mediate the elimination of damaging reactive oxygen species from plants [31]. Moreover, phenolic compounds are widely distributed in plant tissues and play a vital role in plant growth, reproduction, color, flavor, and health benefits [32]. Among them, phenolic acids, flavonoids, and tannins are considered major dietary phenolic compounds [33, 34], offering certain preventive effects against diseases such as cancer and heart disease [35]. Research has shown that peach fruit phenolic compound content varies greatly depending on the species [36]. In this study, the main phenolic compounds, including rutin, apigenin, vanillic acid, chlorogenic acid, caffeic acid, syringic acid, ferulic acid, and *p*-hydroxybenzoic acid, were analyzed and evaluated in olecranon peach. According to Fig. 3, all treatment groups had varying degrees of accumulating effects on eight phenolics in comparison to CK. The accumulation of phenolics such as rutin, apigenin, vanillic acid, ferulic acid, and *p*-hydroxybenzoic acid in olecranon peaches was influenced synergistically by the combination of Nano-Se5 and MT10 treatment, significantly outpacing that of the Nano-Se10 + MT10 treatment group. Specifically, as shown in Fig. 3A, all treatment groups showed a significant increase in rutin content, with the Nano-Se5 + MT10 treatment group accumulating more rutin than the MT10 and Nano-Se10 + MT10 treatment groups by 13.6% and 17.3%, respectively. Figure 3B and C show that the Nano-Se5 + MT10 treatment group had the highest levels of apigenin and vanillic acid accumulation when compared to CK, with apigenin levels 48.3% and 23.0% higher and vanillic acid levels 23.7% and 34.5% higher than in the Nano-Se5 and MT10 individual treatment groups, respectively. There was a 48.4% rise in caffeic acid accumulation in the Nano-Se5 + MT10 treatment group compared to the control group, as shown in Fig. 3D-F, although there was no statistically significant difference in the influence on the chlorogenic acid or syringic acid content. Additionally, Fig. 3G and H demonstrate that the Nano-Se5 + MT10 treatment group exhibited significantly superior accumulation effects for ferulic acid and *p*-hydroxybenzoic acid compared with the other treatment groups. Compared to the control group, the Nano-Se5 + MT10 treatment group showed a 29.4% and 317.3% increase in ferulic acid and *p*-hydroxybenzoic acid content, respectively. The ferulic acid content was higher than that in the Nano-Se5, Nano-Se10, MT10, and Nano-Se10 + MT10 treatment groups by 31.2%, 42.3%, 39.0%, and 34.7%, respectively. The *p*-hydroxybenzoic acid content was 286.7%, 253.3%, and 281.1% higher than that in the Nano-Se5, Nano-Se10, and MT10 treatment groups, respectively, and no significant difference was observed compared to the Nano-Se10 + MT10 treatment group.

### Correlations among parameters analyzed

**Fig. 4:**
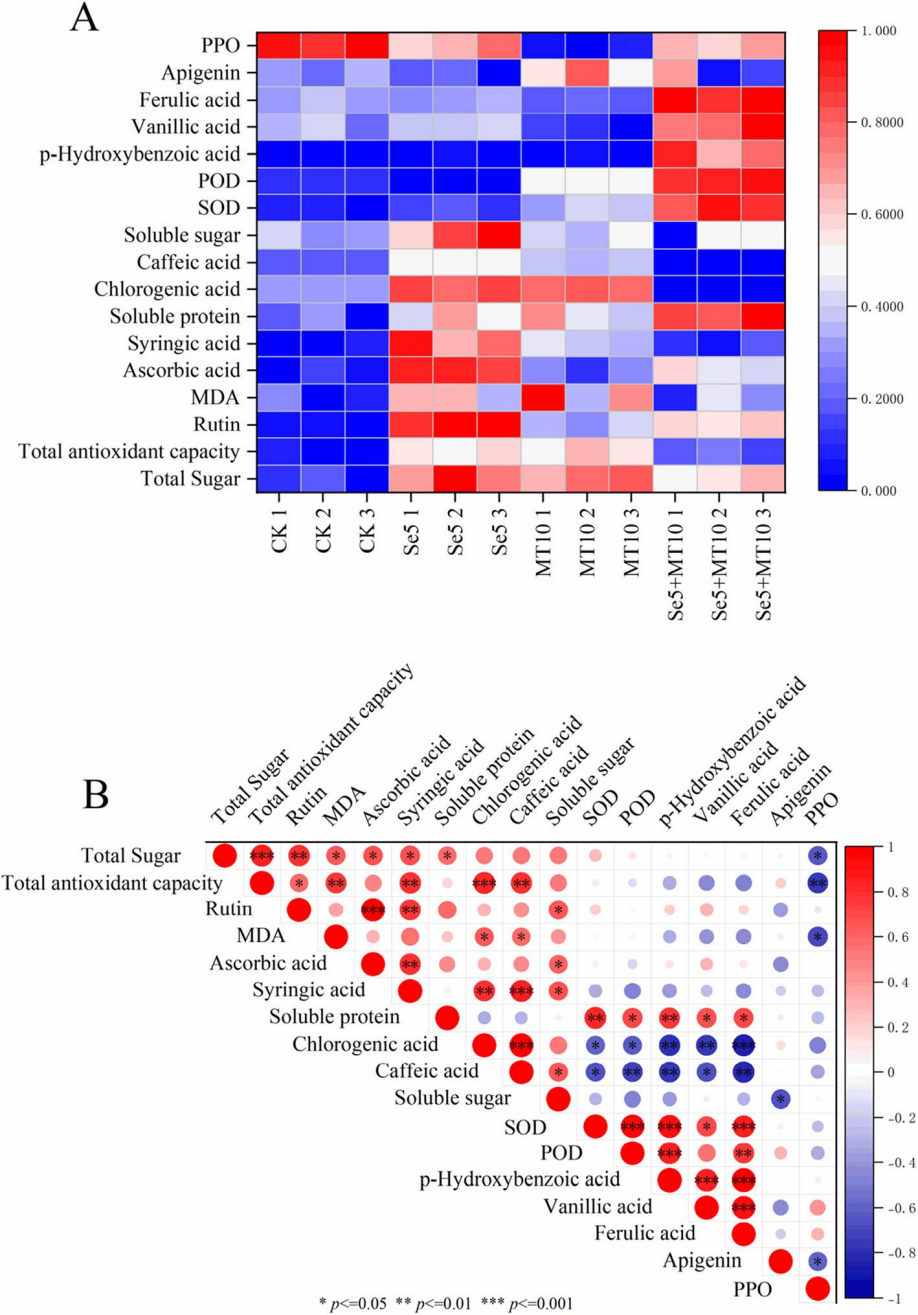
Heat map (**A**) and correlation analysis (**B**) were applied to reveal the relationships between analyzed metabolites in olecranon peach

As shown in Fig. 4A, the application of Nano-Se5 + MT10 was able to induce an increase in phenolic compounds, nutritional quality, and antioxidant-related enzyme activities in olecranon peach and had an inhibitory effect on PPO activity. From Fig. 4B, the parameters analyzed exhibited varying degrees of correlations. Pearson’s correlation coefficients between the indicators analyzed are presented in Supporting Information Table S3. The total antioxidant capacity of olecranon peach showed a significant positive correlation (*P* < 0.05) with secondary metabolites such as rutin (*r* = 0.59), butyric acid (*r* = 0.79), chlorogenic acid (*r* = 0.83) and caffeic acid (*r* = 0.78), and a significant negative correlation (*P* < 0.05) with PPO activity (*r* = -0.74). The ability of a plant to tolerate oxidative stress is determined in large part by its overall antioxidant capacity. Numerous studies have demonstrated that common secondary metabolites in plants, such as rutin, quercetin, chlorogenic acid, caffeic acid, and ferulic acid, exhibit significant antioxidant properties. There was a strong correlation between these secondary metabolites and the total antioxidant capacity [37]. Furthermore, soluble protein showed significant positive correlations (*P* < 0.05) with SOD (*r* = 0.81), POD (*r* = 0.69), *p*-hydroxybenzoic acid (*r* = 0.75), vanillic acid (*r* = 0.67), and ferulic acid (*r* = 0.69). Total sugar exhibited significant positive correlations (*P* < 0.05) with rutin (*r* = 0.79), ascorbic acid (*r* = 0.67), and syringic acid (*r* = 0.68), as well as a highly significant positive correlation (*P* < 0.001) with total antioxidant capacity (*r* = 0.85). The findings suggested a close relationship between the elevation of antioxidant enzymes, phenolic compounds, and non-enzymatic antioxidant molecules in Olecranon peach and the accumulation of nutritional components.

## Discussion

According to studies, plants’ capacity to respond to biotic and abiotic challenges can be improved by activating the antioxidant enzyme system, which can reduce the oxidative damage brought on by an excessive buildup of reactive oxygen species in plants [38]. PPO is an enzyme widely present in fruits that participates in the metabolism of polyphenolic compounds. Its catalytic activity can lead to the oxidation of phenolic substances and the generation of harmful substances, resulting in fruit softening and aging [39]. The regulation of PPO activity has a significant impact on fruit quality and texture, as excessive PPO activity can cause fruit browning [40]. Furthermore, studies have indicated that individual exogenous interventions with 1 mg/L Nano-Se and 0.1 mmol/L MT could upregulate the activity of antioxidant enzymes in fruits and prevent the excessive accumulation of reactive oxygen species, thereby delaying tomato senescence [21] and maintaining the post-harvest quality of peaches [15]. It is interesting to note that this research discovered that the Nano-Se5 + MT10 and Nano-Se10 + MT10 treatment groups further activated superoxide SOD and POD activities in olecranon peach fruits while inhibiting PPO activity. This finding points to a synergistic interaction between Nano-Se and MT in the activation of antioxidant enzymes, attenuation of polyphenol oxidation, enhancement of antioxidant capacity, and maintenance of the dynamic equilibrium between the formation and scavenging of reactive oxygen species in olecranon peach fruits. The results can help olecranon peach retain more of their flavor, quality, and market value by reducing the amount of enzymatic browning and rotting while being transported and stored.

Soluble sugars, soluble proteins, and ascorbic acid play an important role in maintaining plant tolerance to environmental stresses as cellular osmoregulators and antioxidant molecules with the ability to protect cell structure and function, regulate osmotic pressure, and maintain cellular homeostasis [41, 42]. For instance, in the case of drought stress, silicon can improve the water uptake capacity of roots by accumulating amino acids and soluble sugars [43]. The accumulation of cell osmolytes such as soluble sugars, soluble proteins, and proline can enhance wheat’s adaptation to non-biological stresses like drought [44]. Additionally, the accumulation of ascorbic acid alleviates oxidative damage, maintains cellular homeostasis and function, and enhances plant tolerance to non-biological stresses such as salt, cold, and drought [45]. The literature has reported that ascorbic acid reduces the sensitivity of plants to heavy metal stress by scavenging free radicals and protecting cell membrane integrity, promoting the healthy growth of mustard seedlings and plants [46]. Previous studies have demonstrated that selenium influences sugar metabolism in grape berries, promoting fruit growth and sugar accumulation [47], and foliar spraying of 10 mg/L Nano-Se has been found to increase the content of proteins, soluble sugars, chlorophyll, and carotenoids in tea leaves [26]. Notably, this study further revealed that combined intervention with Nano-Se and MT could promote the accumulation of soluble sugars, total sugars, soluble proteins, and ascorbic acid in olecranon peach. It was also observed that the combination of Nano-Se5 + MT10 had a more significant effect on the accumulation of soluble proteins. Correlation analysis further revealed a significant positive correlation between soluble proteins and the activities of SOD and POD in olecranon peach fruits (*P* < 0.05), as well as a significant positive correlation between soluble proteins and total sugars (*P* < 0.05). Therefore, it could be inferred that the combined application of Nano-Se5 + MT10 can activate the antioxidant enzyme system, enhance fruit antioxidant capacity, delay fruit senescence, facilitate the accumulation of soluble proteins and total sugars in olecranon peach fruits, and consequently increase their nutritional value and sweetness. This result is consistent with the findings of previous research on the delayed post-harvest senescence of peaches through the application of 0.1 mmol/L MT [15].

The reduction in PPO activity and the accumulation of phenolic compounds were key factors in enhancing the total antioxidant capacity of olecranon peach [48]. Phenolic compounds, which are significant fruit secondary metabolites, might lower the risk of diabetes and obesity by controlling intestinal flora, reducing inflammation, and lowering fat buildup [49]. Consuming foods rich in polyphenols could neutralize harmful free radicals, mitigate oxidative stress-induced damage to cells and tissues, and reduce the risk of chronic diseases [50]. Furthermore, phenolics play a significant role in reducing the negative impacts of environmental stresses such as salt, heat and cold, drought, and heavy metals on plants by acting as non-enzymatic antioxidants and scavenging reactive oxygen species in plants [51]. One study found that 5 mg/L Nano-Se was used to protect plants from free radical damage and improve the quality of *Siraitia grosvenorii* by promoting the accumulation of phenolic compounds such as apigenin, chlorogenic acid, caffeic acid, butyric acid, ferulic acid and *p*-hydroxybenzoic acid in *Siraitia grosvenorii* [52].

Premature lignification of fruits and vegetables greatly affects their taste and quality, and ferulic acid plays an important regulatory role in plant lignification. It was found that the combined application of 5 mg/L Nano-Se and 20 mg/L lentinan effectively inhibited pea sprout lignification and improved the taste of pea sprouts by significantly elevating the content of ferulic acid, phenylalanine, mustard alcohol, and caffeic alcohol [53]. The accumulation of ferulic acid in olecranon peach may effectively delay fruit lignification, improve fruit taste, and prolong fruit storage life. The present study found that the enhancement of apigenin, ferulic acid, and *p*-hydroxybenzoic acid through the use of Nano-Se alone in olecranon peach was not significant, potentially due to crop species differences. Interestingly, when Nano-Se5 + MT10 was used to treat olecranon peaches, the levels of apigenin, ferulic acid, and *p*-hydroxybenzoic acid increased by 1.5 times, 1.3 times, and 3.9 times, respectively, in comparison to the Nano-Se5 treatment group. Additionally, when compared to the MT10 treatment group, the contents of these compounds were 1.2, 1.4, and 3.8 times higher in the Nano-Se5 + MT10 group.

Correlation analysis revealed a highly significant negative correlation (*P* < 0.01) between PPO activity and total antioxidant capacity (*r* = -0.74), as well as varying degrees of negative correlations with apigenin (*r* = -0.60), chlorogenic acid (*r* = -0.48), caffeic acid and syringic acid. On the other hand, significant positive correlations were observed between vanillic acid (*r* = 0.67), *p*-hydroxybenzoic acid (*r* = 0.75), ferulic acid (*r* = 0.69), and soluble proteins. Therefore, the combined treatment of 5 mg/L Nano-Se and 10 mg/L MT in olecranon peach synergistically enhanced the accumulation of phenolic compounds. This synergistic effect may be attributed to the reduction in PPO activity, leading to decreased oxidative consumption of polyphenols, thereby facilitating the accumulation of phenolic compounds and enhancing the total antioxidant capacity of the fruits. Additionally, it may stimulate the synthesis of osmoregulatory substances such as soluble proteins to counteract various abiotic stresses during fruit development [54]. These findings indicated the potential application value of the combination of Nano-Se and MT in enhancing antioxidant levels, improving environmental stress tolerance, and enhancing fruit quality in Olecranon peach. Moreover, these results provide robust support for further research and application of Nano-Se and MT in agricultural production.

## Conclusion

In conclusion, our findings showed that different ratios of Nano-Se and MT might be used to improve the antioxidant capacity and nutritional value of olecranon peaches. The combination of Nano-Se5 + MT10 had the greatest impact on the improvement in olecranon peach quality when compared to the control. By decreasing PPO activity and increasing SOD and POD activity, the combination of Nano-Se and MT increased the total antioxidant capacity of olecranon peaches. This facilitated the accumulation of soluble protein and total sugars in fruits, which may have reduced oxidative stress caused by environmental stress during olecranon peach growth and enhanced the flavor and sweetness of fruits. In addition, the combination of Nano-Se5 + MT10 treatment may contribute in some way to avoiding fruit senescence, softening, and browning as well as extending fruit shelf life. However, more investigation is needed to clarify the precise mechanisms. The findings of this study offer both technical justification and proof for the sensible application of Nano-Se and MT in the cultivation of olecranon peaches to increase quality and productivity. Along with addressing issues with transportation and preservation, they also provide fresh perspectives and strategies for improving the peach fruit’s capacity to withstand stress.

## Supplementary Information

**Additional file 1: Table S1.** The gradient elution program for phenolic compounds. **Table S2.** Mass parameters for phenolic compounds analysis. **Table S3.** Pearson’s correlation coefficients (*r*) between analyzed metabolites in Olecranon peaches.

## Data Availability

The authors confirm that the data supporting the findings of this study are available within the article and its supplementary materials.

## References

[1] Niu Y, Deng J, Xiao Z, Zhu J. Characterization of the major aroma-active compounds in peach (*Prunus persica* L. Batsch) by gas chromatography–olfactometry, flame photometric detection and molecular sensory science approaches. Food Res Int. 2021;147:110457. 34399457 10.1016/j.foodres.2021.110457

[2] Zhu J-K. Abiotic stress signaling and responses in plants. Cell. 2016;167:313–24. 27716505 10.1016/j.cell.2016.08.029 PMC5104190

[3] Neme K, Nafady A, Uddin S, Tola YB. Application of nanotechnology in agriculture, postharvest loss reduction and food processing: food security implication and challenges. Heliyon. 2021;7:e08539. 34934845 10.1016/j.heliyon.2021.e08539 PMC8661015

[4] Wu C, Hao W, Yan L, Zhang H, Zhang J, Liu C, Zheng L. Postharvest melatonin treatment enhanced antioxidant activity and promoted GABA biosynthesis in yellow-flesh peach. Food Chem. 2023;419:136088. 37023675 10.1016/j.foodchem.2023.136088

[5] de Queiroz AR, Hines C, Brown J, Sahay S, Vijayan J, Stone JM, Bickford N, Wuellner M, Glowacka K, Buan NR, Roston RL. The effects of exogenously applied antioxidants on plant growth and resilience. Phytochem Rev. 2023;22:407–47.

[6] Cardinali DP, Srinivasan V, Brzezinski A, Brown GM. Melatonin and its analogs in insomnia and depression. J Pineal Res. 2012;52:365–75. 21951153 10.1111/j.1600-079X.2011.00962.x

[7] Dubbels R, Reiter RJ, Klenke E, Goebel A, Schnakenberg E, Ehlers C, Schiwara HW, Schloot W. Melatonin in edible plants identified by radioimmunoassay and by high-performance liquid chromatography-mass spectrometry. J Pineal Res. 1995;18:28–31. 7776176 10.1111/j.1600-079x.1995.tb00136.x

[8] Castanares JL, Bouzo CA. Effect of exogenous melatonin on seed germination and seedling growth in melon (*Cucumis melo* L.) under salt stress. Hortic Plant J. 2019;5:79–87.

[9] Bahcesular B, Yildirim ED, Karacocuk M, Kulak M, Karaman S. Seed priming with melatonin effects on growth, essential oil compounds and antioxidant activity of basil (*Ocimum basilicum* L.) under salinity stress. Ind Crops Prod. 2020;146:112165.

[10] Chen Q, Hou SY, Pu XJ, Li XM, Li RR, Yang Q, Wang XJ, Guan M, Rengel Z. Dark secrets of phytomelatonin. J Exp Bot. 2022;73:5828–39. 35522068 10.1093/jxb/erac168

[11] Huang B, Chen Y-E, Zhao Y-Q, Ding C-B, Liao J-Q, Hu C, Zhou L-J, Zhang Z-W, Yuan S, Yuan M. Exogenous melatonin alleviates oxidative damages and protects photosystem II in maize seedlings under drought stress. Front Plant Sci. 2019;10:677. 31178885 10.3389/fpls.2019.00677 PMC6543012

[12] Jiao X, Deng B, Zhang L, Gao Z, Feng Z, Wang R. Melatonin and 1-Methylcyclopropene improve the postharvest quality and antioxidant capacity of ‘Youhou’ sweet persimmons during cold storage. Int J Fruit Sci. 2022;22:809–25.

[13] Wei W, Li Q-T, Chu Y-N, Reiter RJ, Yu X-M, Zhu D-H, Zhang W-K, Ma B, Lin Q, Zhang J-S, Chen S-Y. Melatonin enhances plant growth and abiotic stress tolerance in soybean plants. J Exp Bot. 2015;66:695–707. 25297548 10.1093/jxb/eru392 PMC4321538

[14] Madebo MP, Zheng Y, Jin P. Melatonin-mediated postharvest quality and antioxidant properties of fresh fruits: a comprehensive meta-analysis. Compr Rev Food Sci F. 2022;21:3205–26. 10.1111/1541-4337.12961 35621156

[15] Gao H, Zhang ZK, Chai HK, Cheng N, Yang Y, Wang DN, Yang T, Cao W. Melatonin treatment delays postharvest senescence and regulates reactive oxygen species metabolism in peach fruit. Postharvest Biol Tec. 2016;118:103–10.

[16] Shah HMS, Singh Z, Ul Hasan M, Afrifa-Yamoah E, Woodward A. Preharvest melatonin application alleviates red drupelet reversion, improves antioxidant potential and maintains postharvest quality of ‘Elvira’ blackberry. Postharvest Biol Tec. 2023;203:112418.

[17] Usman M, Farooq M, Wakeel A, Nawaz A, Cheema SA, Rehman HU, Ashraf I, Sanaullah M. Nanotechnology in agriculture: current status, challenges and future opportunities. Sci Total Environ. 2020;721:137778. 32179352 10.1016/j.scitotenv.2020.137778

[18] Chauhan R, Awasthi S, Srivastava S, Dwivedi S, Pilon-Smits EAH, Dhankher OP, Tripathi RD. Understanding selenium metabolism in plants and its role as a beneficial element. Crit Rev Environ Sci Tec. 2019;49:1937–58.

[19] Zhang J-S, Gao X-Y, Zhang L-D, Bao Y-P. Biological effects of a nano red elemental selenium. BioFactors. 2001;15:27–38. 11673642 10.1002/biof.5520150103

[20] Li D, Zhou C, Zhang J, An Q, Wu Y, Li J-Q, Pan C. Nanoselenium foliar applications enhance the nutrient quality of pepper by activating the capsaicinoid synthetic pathway. J Agric Food Chem. 2020;68:9888–95. 32809823 10.1021/acs.jafc.0c03044

[21] Liu R, Deng Y, Zheng M, Liu Y, Wang Z, Yu S, Nie Y, Zhu W, Zhou Z, Diao J. Nano selenium repairs the fruit growth and flavor quality of tomato under the stress of penthiopyrad. Plant Physiol Biochem. 2022;184:126–36. 35640519 10.1016/j.plaphy.2022.05.026

[22] Kang L, Wu Y, Zhang J, An Q, Zhou C, Li D, Pan C. Nano-selenium enhances the antioxidant capacity, organic acids and cucurbitacin B in melon (*Cucumis melo* L.) plants. Ecotox Environ Safe. 2022;241:113777. 10.1016/j.ecoenv.2022.113777 35738099

[23] Kang L, Wu Y, Jia Y, Chen Z, Kang D, Zhang L, Pan C. Nano-selenium enhances melon resistance to *Podosphaera xanthii* by enhancing the antioxidant capacity and promoting alterations in the polyamine, phenylpropanoid and hormone signaling pathways. J Nanobiotechnol. 2023;21:377. 10.1186/s12951-023-02148-y PMC10577987 37845678

[24] Jia Y, Kang L, Wu Y, Zhou C, Cai R, Zhang H, Li J, Chen Z, Kang D, Zhang L, Pan C. Nano-selenium foliar intervention-induced resistance of cucumber to Botrytis cinerea by activating jasmonic acid biosynthesis and regulating phenolic acid and cucurbitacin. Pest Manag Sci. 2023. 10.1002/ps.7784. 10.1002/ps.7784 37733166

[25] Zhou C, Zhang J, Miao P, Dong Q, Lin Y, Li D, Pan C. Novel finding on how melatonin and nanoselenium alleviate 2,4-D butylate stress in wheat plants. J Agric Food Chem. 2023;71:12943–57. 37622422 10.1021/acs.jafc.3c03109

[26] Li D, Zhou C, Zou N, Wu Y, Zhang J, An Q, Li J-Q, Pan C. Nanoselenium foliar application enhances biosynthesis of tea leaves in metabolic cycles and associated responsive pathways. Environ Pollut. 2021;273:116503. 33486255 10.1016/j.envpol.2021.116503

[27] Ali S, Khan AS, Malik AU, Shahid M. Effect of controlled atmosphere storage on pericarp browning, bioactive compounds and antioxidant enzymes of litchi fruits. Food Chem. 2016;206:18–29. 27041293 10.1016/j.foodchem.2016.03.021

[28] Jiang Y, Duan X, Joyce D, Zhang Z, Li J. Advances in understanding of enzymatic browning in harvested litchi fruit. Food Chem. 2004;88:443–6.

[29] Yan J, Luo Z, Ban Z, Lu H, Li D, Yang D, Aghdam MS, Li L. The effect of the layer-by-layer (LBL) edible coating on strawberry quality and metabolites during storage. Postharvest Biol Tec. 2019;147:29–38.

[30] Serna-Escolano V, Martínez-Romero D, Giménez MJ, Serrano M, García-Martínez S, Valero D, Valverde JM, Zapata PJ. Enhancing antioxidant systems by preharvest treatments with methyl jasmonate and salicylic acid leads to maintain lemon quality during cold storage. Food Chem. 2021;338:128044. 32932092 10.1016/j.foodchem.2020.128044

[31] Samec D, Karalija E, Sola I, Vujcic Bok V, Salopek-Sondi B. The role of polyphenols in abiotic stress response: the influence of molecular structure. Plants-Basel. 2021;10:118. 33430128 10.3390/plants10010118 PMC7827553

[32] Alasalvar C, Grigor JM, Zhang DL, Quantick PC, Shahidi F. Comparison of volatiles, phenolics, sugars, antioxidant vitamins, and sensory quality of different colored carrot varieties. J Agric Food Chem. 2001;49:1410–6. 11312873 10.1021/jf000595h

[33] King A, Young G. Characteristics and occurrence of phenolic phytochemicals. J Am Diet Assoc. 1999;99:213–8. 9972191 10.1016/S0002-8223(99)00051-6

[34] Tain Y-L, Hsu C-N. Novel insights on dietary polyphenols for prevention in early-life origins of hypertension: a review focusing on preclinical animal models. Int J Mol Sci. 2022;23:6620. 35743061 10.3390/ijms23126620 PMC9223825

[35] Koch W. Dietary polyphenolsImportant non-nutrients in the prevention of chronic noncommunicable diseases. A systematic review. Nutrients. 2019;11:1039. 31075905 10.3390/nu11051039 PMC6566812

[36] Wu H, Xu Y, Wang H, Miao Y, Li C, Zhao R, Shi X, Wang B. Physicochemical characteristics, antioxidant activities, and aroma compound analysis of seven peach cultivars (*Prunus persica* L. Batsch) in Shihezi, Xinjiang. Foods. 2022;11:2944. 36230020 10.3390/foods11192944 PMC9563965

[37] Zugic A, Dordevic S, Arsic I, Markovic G, Zivkovic J, Jovanovic S, Tadic V. Antioxidant activity and phenolic compounds in 10 selected herbs from Vrujci Spa, Serbia. Ind Crops Prod. 2014;52:519–27.

[38] Liu T, Li T, Zhang L, Li H, Liu S, Yang S, An Q, Pan C, Zou N. Exogenous salicylic acid alleviates the accumulation of pesticides and mitigates pesticide-induced oxidative stress in cucumber plants (*Cucumis sativus*). Ecotox Environ Safe. 2021;208:111654. 10.1016/j.ecoenv.2020.111654 33396168

[39] Toivonen PMA, Brummell DA. Biochemical bases of appearance and texture changes in fresh-cut fruit and vegetables. Postharvest Biol Tec. 2008;48:1–14.

[40] Wei Y, Yu N, Zhu Y, Hao J, Shi J, Lei Y, Gan Z, Jia G, Ma C, Sun A. Exploring the biochemical properties of three polyphenol oxidases from blueberry (*Vaccinium corymbosum* L.). Food Chem. 2021;344:128678. 33267982 10.1016/j.foodchem.2020.128678

[41] Liu Y, He Z, Xie Y, Su L, Zhang R, Wang H, Li C, Long S. Drought resistance mechanisms of *Phedimus aizoon L*. Sci Rep. 2021;11:13600. 34193957 10.1038/s41598-021-93118-7 PMC8245562

[42] Pandey S, Fartyal D, Agarwal A, Shukla T, James D, Kaul T, Negi YK, Arora S, Reddy MK. Abiotic stress tolerance in plants: myriad roles of ascorbate peroxidase. Front Plant Sci. 2017;8:581. 28473838 10.3389/fpls.2017.00581 PMC5397514

[43] Sonobe K, Hattori T, An P, Tsuji W, Eneji AE, Kobayashi S, Kawamura Y, Tanaka K, Inanaga S. Effect of silicon application on sorghum root responses to water stress. J Plant Nutr. 2010;34:71–82.

[44] Cheraghi M, Motesharezadeh B, Mousavi SM, Ma QF, Ahmadabadi Z. Silicon (Si): a regulator nutrient for optimum growth of wheat under salinity and drought stresses- a review. J Plant Growth Regul. 2023;42:5354–78.

[45] Bilska K, Wojciechowska N, Alipour S, Kalemba EM. Ascorbic acid-the little-known antioxidant in woody plants. Antioxidants. 2019;8:645. 31847411 10.3390/antiox8120645 PMC6943661

[46] Sharma R, Bhardwaj R, Thukral AK, Al-Huqail AA, Siddiqui MH, Ahmad P. Oxidative stress mitigation and initiation of antioxidant and osmoprotectant responses mediated by ascorbic acid in *Brassica juncea* L. subjected to copper (II) stress. Ecotox Environ Safe. 2019;182:109436. 10.1016/j.ecoenv.2019.109436 31325808

[47] Zhu S, Liang Y, An X, Kong F, Gao D, Yin H. Changes in sugar content and related enzyme activities in table grape (*Vitis vinifera* L.) in response to foliar selenium fertilizer. J Sci Food Agr. 2017;97:4094–102. 28211621 10.1002/jsfa.8276

[48] Mei Y, Sun H, Du G, Wang X, Lyu D. Exogenous chlorogenic acid alleviates oxidative stress in apple leaves by enhancing antioxidant capacity. Sci Hortic. 2020;274:109676.

[49] Chen L, Pu Y, Xu Y, He X, Cao J, Ma Y, Jiang W. Anti-diabetic and anti-obesity: efficacy evaluation and exploitation of polyphenols in fruits and vegetables. Food Res Int. 2022;151:111202. 10.1016/j.foodres.2022.111202 35761524

[50] Gasmi A, Mujawdiya PK, Noor S, Lysiuk R, Darmohray R, Piscopo S, Lenchyk L, Antonyak H, Dehtiarova K, Shanaida M, Polishchuk A, Shanaida V, Peana M, Bjørklund G. Polyphenols in metabolic diseases. Molecules. 2022;27:6280. 36234817 10.3390/molecules27196280 PMC9570923

[51] Zagoskina NV, Zubova MY, Nechaeva TL, Kazantseva VV, Goncharuk EA, Katanskaya VM, Baranova EN, Aksenova MA. Polyphenols in plants: structure, biosynthesis, abiotic stress regulation, and practical applications (Review). Int J Mol Sci. 2023;24:13874. 37762177 10.3390/ijms241813874 PMC10531498

[52] Zhou C, Zhang J, Wu Y, Cheng H, Pang Q, Xiao Y, Li D, Pan C. Metabolomic analysis on the mechanism of nanoselenium biofortification improving the siraitia grosvenorii nutritional and health value. Foods. 2022;11:3019. 36230095 10.3390/foods11193019 PMC9564208

[53] Lin Y, Zhou C, Li D, Wu Y, Dong Q, Jia Y, Yu H, Miao P, Pan C. Integrated non-targeted and targeted metabolomics analysis reveals the mechanism of inhibiting lignification and optimizing the quality of pea sprouts by combined application of nano-selenium and lentinans. J Sci Food Agr. 2023;103:5096–107. 36974656 10.1002/jsfa.12579

[54] Tahjib-Ul-Arif M, Zahan MI, Karim MM, Imran S, Hunter CT, Islam MS, Mia MA, Hannan MA, Rhaman MS, Hossain MA, Brestic M, Skalicky M, Murata Y. Citric acid-mediated abiotic stress tolerance in plants. Int J Mol Sci. 2021;22:7235. 34281289 10.3390/ijms22137235 PMC8268203

