# Supplementary material for: Enhanced antioxidant activity and quality of olecranon peach fruits (Prunus persica L.) through synergistic application of exogenous nano-selenium and melatonin
Source: Crop Health. 2023 Dec 5;1(1):17. doi: 10.1007/s44297-023-00017-6 (PMC12825980; doi:10.1007/s44297-023-00017-6)
Supplement: Supplementary file 1 — Additional file 1: Table S1. The gradient elution program for phenolic compounds. Table S2. Mass parameters for phenolic compounds analysis. Table S3. Pearson’s correlation coefficients (r) between analyzed metabolites in Olecranon peaches. [file 44297_2023_17_MOESM1_ESM.docx]

**Enhanced Antioxidant Activity and Quality of Olecranon Peach Fruits (*Prunus persica* L.) through Synergistic Application of Exogenous Nano-Selenium and Melatonin**

Peijuan Miao ^1^, Qinyong Dong ^1^, Chunran Zhou ^1^, Dong Li ^2^, Huan Yu ^1^, Yongxi Lin ^1^, Yangliu Wu ^1^, Canping Pan ^1, *^

^1^ Key Laboratory of Tropical Fruits and Vegetables Quality and Safety for State Market Regulation, College of Science, China Agricultural University, Haikou 570311, China；

^2^ Key Laboratory of Green Prevention and Control of Tropical Plant Diseases and Pests, Ministry of Education, College of Plant Protection, Hainan University, Haikou, Hainan 570228, People’s Republic of China

*Corresponding author

E-mail address: [canpingp@cau.edu.cn](mailto:canpingp@cau.edu.cn)

Total number of pages: 3

Total number of tables: 3

**SUPPORTING TABLES**

**Table S1.** The gradient elution program for phenolic compounds

**Table S2.** Mass parameters for phenolic compounds analysis

**Table S3.** Pearson’s correlation coefficients (*r*) between analyzed metabolites in Olecranon peaches

**Table S1.** The gradient elution program for phenolic compounds

| Time (min) | Mobile phase A (%) | Mobile phase B (%) | Flow rate (mL/min) |
| --- | --- | --- | --- |
| 0.00 | 95.00 | 5.00 | 0.400 |
| 5.00 | 0.00 | 100.00 | 0.400 |
| 6.00 | 0.00 | 100.00 | 0.400 |
| 6.01 | 95.00 | 5.00 | 0.400 |
| 7.30 | 95.00 | 5.00 | 0.400 |

Note: A, acetonitrile, B, 0.1% (v:v) formic acid aqueous solution.

**Table S2.** Mass parameters for phenolic compounds analysis

| Compound | Retention time/min | Precursor ion, m/z | Product ion, m/z | Cone voltage/V | Collision energies/eV |
| --- | --- | --- | --- | --- | --- |
| Rutin | 1.516 | 609.2 | 300/270.9 | 200 | 35 |
| Apigenin | 2.312 | 269.1 | 117/225 | 150 | 40 |
| Vanillic acid | 1.229 | 167 | 152/123 | 70 | 10 |
| Chlorogenic acid | 1.548 | 353.1 | 191.1/127.1 | 65 | 15 |
| Caffeic acid | 1.366 | 179 | 135/79.1 | 160 | 15 |
| Syringic acid | 1.759 | 197 | 182/167 | 70 | 10 |
| Ferulic Acid | 2.070 | 193 | 134/177.8 | 90 | 18 |
| 4-Hydroxybenzoic acid | 1.607 | 137 | 93.1/65.1 | 70 | 15 |

**Table S3.** Pearson’s correlation coefficients (*r*) between analyzed metabolites in Olecranon peaches

|  | X_1_ | X_2_ | X_3_ | X_4_ | X_5_ | X_6_ | X_7_ | X_8_ | X_9_ | X_10_ | X_11_ | X_12_ | X_13_ | X_14_ | X_15_ | X_16_ | X_17_ |
| --- | --- | --- | --- | --- | --- | --- | --- | --- | --- | --- | --- | --- | --- | --- | --- | --- | --- |
| X_1_ | 1.00 |  |  |  |  |  |  |  |  |  |  |  |  |  |  |  |  |
| X_2_ | 0.85 | 1.00 |  |  |  |  |  |  |  |  |  |  |  |  |  |  |  |
| X_3_ | 0.79 | 0.59 | 1.00 |  |  |  |  |  |  |  |  |  |  |  |  |  |  |
| X_4_ | 0.64 | 0.74 | 0.37 | 1.00 |  |  |  |  |  |  |  |  |  |  |  |  |  |
| X_5_ | 0.67 | 0.50 | 0.97 | 0.31 | 1.00 |  |  |  |  |  |  |  |  |  |  |  |  |
| X_6_ | 0.68 | 0.79 | 0.76 | 0.56 | 0.78 | 1.00 |  |  |  |  |  |  |  |  |  |  |  |
| X_7_ | 0.58 | 0.19 | 0.57 | 0.24 | 0.46 | 0.08 | 1.00 |  |  |  |  |  |  |  |  |  |  |
| X_8_ | 0.52 | 0.83 | 0.31 | 0.63 | 0.32 | 0.79 | -0.31 | 1.00 |  |  |  |  |  |  |  |  |  |
| X_9_ | 0.52 | 0.78 | 0.41 | 0.59 | 0.44 | 0.85 | -0.29 | 0.98 | 1.00 |  |  |  |  |  |  |  |  |
| X_10_ | 0.52 | 0.51 | 0.66 | 0.40 | 0.61 | 0.66 | 0.06 | 0.53 | 0.64 | 1.00 |  |  |  |  |  |  |  |
| X_11_ | 0.28 | -0.08 | 0.22 | -0.03 | 0.08 | -0.32 | 0.81 | -0.62 | -0.65 | -0.28 | 1.00 |  |  |  |  |  |  |
| X_12_ | 0.14 | -0.14 | -0.04 | -0.05 | -0.18 | -0.47 | 0.69 | -0.63 | -0.71 | -0.48 | 0.95 | 1.00 |  |  |  |  |  |
| X_13_ | 0.06 | -0.34 | 0.20 | -0.30 | 0.13 | -0.38 | 0.75 | -0.78 | -0.78 | -0.37 | 0.92 | 0.85 | 1.00 |  |  |  |  |
| X_14_ | -0.03 | -0.45 | 0.32 | -0.40 | 0.29 | -0.25 | 0.67 | -0.76 | -0.68 | -0.07 | 0.70 | 0.55 | 0.86 | 1.00 |  |  |  |
| X_15_ | -0.06 | -0.47 | 0.18 | -0.43 | 0.13 | -0.42 | 0.69 | -0.85 | -0.82 | -0.30 | 0.84 | 0.76 | 0.97 | 0.93 | 1.00 |  |  |
| X_16_ | 0.06 | 0.21 | -0.38 | 0.08 | -0.43 | -0.18 | -0.07 | 0.14 | -0.02 | -0.65 | 0.08 | 0.29 | -0.01 | -0.44 | -0.19 | 1.00 |  |
| X_17_ | -0.63 | -0.74 | -0.09 | -0.70 | 0.03 | -0.27 | -0.28 | -0.48 | -0.34 | 0.05 | -0.24 | -0.34 | 0.10 | 0.44 | 0.29 | -0.60 | 1.00 |

Note: X_1_, Total sugar; X_2_, Total antioxidant capacity; X_3_, Rutin; X_4_, MDA; X_5_, Ascorbic acid; X_6_, Syringic acid; X_7_, Soluble protein; X_8_, Chlorogenic acid; X_9_, Caffeic acid; X_10_, Soluble sugar; X_11_, SOD; X_12_, POD; X_13_, *p*-Hydroxybenzoic acid; X_14_, Vanillic acid; X_15_, Ferulic acid; X_16_, Apigenin; X_17_, PPO.
